# Representations of cycling in metropolitan newspapers - changes over time and differences between Sydney and Melbourne, Australia

**DOI:** 10.1186/1471-2458-10-371

**Published:** 2010-06-25

**Authors:** Chris Rissel, Catriona Bonfiglioli, Adrian Emilsen, Ben J Smith

**Affiliations:** 1 Health Promotion Service, Sydney South West Area Health Service, University of Sydney, King George V Building, Missenden Road, Camperdown 2050, Australia; 2 Faculty of Arts and Social Sciences, University of Technology, Sydney, Australia; 3 Office of Facilities Management, Macquarie University, Sydney, Australia; 4 Department of Health Social Science, Monash University, Melbourne, Australia

## Abstract

**Background:**

Cycling is important for health, transport, environmental and economic reasons. Newspaper reporting of cycling reflects and can influence public and policy maker attitudes towards resource allocation for cycling and cycling infrastructure, yet such coverage has not been systematically examined.

**Methods:**

The Factiva electronic news archive was searched for articles referring to cycling published in four major metropolitan newspapers - two in Sydney and two in Melbourne, Australia, in the years from 1998 until 2008. After excluding articles not about cycling, there were 61 articles published in 1998, 45 in 1999, 51 in 2003, 82 in 2007 and 87 in 2008. Each article was coded for positive or negative orientation, and for framing of cyclists and cycling. Inter-rater reliability was calculated on a sample of 30 articles.

**Results:**

Over the past decade there has been an increase in the reporting of cycling in the major newspapers in Sydney and Melbourne (from 106 in 1998/99 to 169 stories in 2007/08), with a significant increase in reporting of cycling in Melbourne, from 49 to 103 stories (p = 0.04). Recent reporting of cycling was generally positive (47% of articles, compared with 30% of articles which were negative) and focused on benefits such as health and the environment. Three quarters of negative stories involved injury or death of a cyclist. The Sydney based *The Daily Telegraph *reported the most negative stories (n = 60). We found positive framing of 'cycling' was more widespread than negative, whereas framing of 'cyclists' was more negative than positive.

**Conclusions:**

Quantity of reporting of cycling varies over time and by newspaper, and even between newspapers in different cities owned by the same media company. News coverage appears to reflect developments in the different cities, with increases in positive reporting of cycling in Melbourne following increases in cycling in that city. Negative cycling newspaper stories may deter people from considering cycling as a transport option, but real physical or political improvements to the cycling environment may be necessary before coverage becomes more positive.

## Background

Cycling is the fourth most popular recreation in Australia, with bicycles increasingly used as transport across the country [1]. About a million new bicycles are sold in Australia each year, and cycling is a form of regular physical activity that is accessible to people of all ages and confers substantial health benefits [1].

Despite its popularity, the growth of cycling for recreation and transport is potentially limited by the availability of infrastructure that enables it to be performed easily and safely. Of interest to advocates of cycling, therefore, are the perceptions of the public and of policy makers towards this activity, because of the bearing these perspectives have on the planning and investments needed to support cycling. Researching the portrayal of cycling in the media can shed light on the climate of beliefs and values in which policies that support or hinder cycling are made. News media coverage has particular salience because it can shape public understandings of issues and influence policy and behaviours [2,3].

The limited research undertaken on the news reporting of cycling has identified both positive and negative framings of cycling that have shifted across time within different societies. Following the emergence of the bicycle in the mid 19^th ^century, the media drew attention to the wonder and amazement that was generated by this new machine. Early users of the bicycle were also viewed in some quarters with scorn, with some newspapers portraying cyclists as hoodlums of ill manner.

The 1890s have been described as the 'golden era' of cycling [4]. As bicycles became cheaper and more user-friendly, interest grew to the point where entire sections of newspapers were dedicated to bicycle news. According to Hammond [4], the economic benefits generated by bicycle advertising swiftly transformed both press and public attitudes:

Bicycle interests allocated thousands of dollars annually to instruct the public about the lightness, swiftness, strength and beauty of their product... In return, by running editorials and regular columns on the wheel, plus special articles by bicycle enthusiasts and medical men, the press and magazines made cycling an increasingly more discussed and respectable activity.

In the early 20^th ^century, media reporting of cycling declined as the motor car became more popular even though the actual number of cyclists continued to grow. News reporting focused more on cycling as sport and less on its utilitarian values [5]. Some researchers have also noted that in car-centric societies, images of cyclists are dominated by negative representations. According to Bogdanowicz [6]:

Where the bicycle is featured in UK newsprint it's frequently characterised as a mode of transport for eccentrics or 'tree-huggers'. Columns or programmes about "Lycra clad fascists" and "Lycra nazis" are re-cycled on a regular basis by newspaper columnists and radio shock-jocks.

Bogdanowicz [6] and Horton [5] argue that there has been an emergence in recent years of biases against cycling in the news media due to the economic and cultural dominance of the automotive industry and their advertising power within the news media. More positive reporting of cycling, however, has also been documented. Fincham, for example, reports that many positive stories have an 'emphasis on the health benefits of cycling coupled with the perception of freedom that is associated with cycling' [7].

Because of the importance of cycling, in health, transport, environmental and economic terms, further research about the way that this has been framed in news media in recent years is needed. The news media, and newspapers particularly, warrant investigation because they are a primary source of information that can reflect and reinforce community attitudes. The news media also play an important agenda setting function, by influencing what people think about [2] and attitudes to issues [3]. The higher up the news agenda an issue is, the more likely it is to be seen as important by the public [2]. Thus the prominence and type of news coverage cycling receives is likely to be shaping public understandings of cyclists and cycling and is of importance to the uptake of cycling and to public policy support for cycling.

This study aims to investigate how cycling and cyclists have been represented in Australian newspapers over the past decade. It examines the frequency of cycling related news stories in major metropolitan newspapers, the way cycling and cyclists are framed by news stories, and variations in the representations of cycling over time, by type of newspaper, and by geographical location.

## Methods

Our study draws on social construction of reality theory which explains how individuals use news texts and other information to make sense of the world and uses content analysis to examine the nature of newspaper coverage of cycling and the dominant frames [3,8] used to portray cycling and cyclists. Initial frames were identified from a focus group study on perceptions of cyclists [9], however coding allowed for new frames to be identified in the newspaper sample. This study was designed to discover how the two separate but related constructs of 'cycling' and 'cyclists' are portrayed in the news and whether there are differences between Sydney and Melbourne newspapers.

### Sample

The news coverage analysed was limited to newspaper articles from *The Sydney Morning Herald*, *The Daily Telegraph *(Sydney), *The Age *(Melbourne) and The *Herald Sun *(Melbourne). These are the major newspapers in the two largest cities in Australia, with a potential reach of almost half the Australian population. *The Sydney Morning Herald *and *The Age *are broadsheet newspapers and both published by Fairfax Media. The two tabloid newspapers, *The Daily Telegraph *(Sydney), and the *Herald Sun *(Melbourne), are owned by News Ltd.

The Factiva electronic news archive was searched for articles referring to cycling [search terms Rst = (AGEE OR HERSUN OR DAITEL OR SMHH) AND hd = (cycling OR cyclist OR bicycl* OR bike)] published in selected publications in the years 1998, 1999, 2003, 2007, 2008. These years were selected to cover a decade, and to provide a data point in the middle to allow examination of any trend. This search generated 104 articles in 1998, 73 in 1999, 88 in 2003, 121 in 2007 and 120 in 2008. After viewing the articles, a number were removed as they did not meet eligibility criteria (for example, they were stories about motorbikes, stationary exercise bikes, non-news items). Opinion pieces were excluded as we aimed to focus on news and features, rather than the often extreme views presented in opinion pieces. Editorials, sports news, letters to the editor and news in brief items were also excluded. After exclusions the samples for each year were 61 articles in 1998, 45 in 1999, 51 in 2003, 82 in 2007 and 87 in 2008.

### Inter-rater reliability

One coder reviewed all downloaded articles, and coded all eligible articles according to the coding frame. A second coder (BS) reviewed a random sample of 30 (approximately 10%) of these articles. Inter-rater reliability was assessed using Cohen's Kappa.

### Analysis

To determine the change in the proportion of articles about cycling in each newspaper over time, and to assess change in the proportion of articles that were positive or negative about cycling in each city, articles from 1998 and 1999 were treated as one period, as were articles from 2007 and 2008. The chi-square statistic was used to test whether the proportions were significantly different over the 10 year period. Because the total number of framings of cycling and cyclists within articles was greater than the number of articles, the analysis of the frequency and proportion of different framings was undertaken separately for each of the five data collection years.

## Results

Inter-rater agreement on coding was good, with a Cohen's Kappa of 0.77 for identification of the positive and negative frames of cycling and cyclists within articles.

### Trends in news coverage of cycling

There was an overall increase in the frequency of newspaper stories about cycling in Melbourne and Sydney from 1998-99 (n = 106) to 2007-08 (n = 169) and slightly more frequent reporting of cycling in Melbourne (54.3%) than in Sydney (45.7%). Over the 10-year period the number of cycling-related stories significantly increased from 49 to 103 stories in Melbourne (χ^2 ^= 6.37, p = 0.04) (see Table 1). The total number of all articles published by these four newspapers in our study years increased from about 130,000 in 1998 to about 145,000 in 2008.

**Table 1:** Number and per cent of news stories per year concerning cycling by city and newspaper, 1998-2008

	Sydney						Melbourne						
			
	*Daily Telegraph*		SMH*		*All*		*Herald Sun*		*The Age*		*All*		Total
	
	N	%	N	%	N	%	N	%	N	%	N	%	
1998-99	45	42.5	12	11.3	57	53.8	36	34.0	13	12.3	49	46.2	106
2003	19	37.3	7	13.7	26	51.0	15	29.4	10	19.6	25	49.0	51
2007-08	40	23.7	26	15.4	66	39.1	56	33.1	47	27.8	103	61.0	169
**Total**	104	31.9	45	13.8	149	45.7	107	32.8	70	21.5	177	54.3	326

*Sydney Morning Herald

Due to the greater increase in cycling news stories in Melbourne than Sydney, there was a decline in the relative proportion of items that were published in Sydney newspapers in the study period. As shown in Table 1, this was due to less reporting of cycling by *The Daily Telegraph *(with a small increase in *The Sydney Morning Herald*). There was double the number of articles about cycling in 2007-08 in Melbourne in *The Age *compared to earlier periods.

Table 2 shows that there was an increase in stories about cycling that were classified as positive over the last decade, with the largest increase in the last few years. This pattern is mirrored by a decrease in overall negative stories. There was no change in the frequency of neutral stories. Overall, there were slightly more positive news stories about cycling than negative stories.

**Table 2:** Number and per cent of positive and negative reporting of cycling by city and over time

	Positive		Negative	
	(N = 128)		(N = 118)	
	N	%	N	%
**City**				
Melbourne	81	45.8	51	28.8
Sydney	47	31.5	67	45.0
	χ^2 ^= 6.86, p < .01		χ^2 ^= 9.14, p < .01	
**Year**				
1998-99	32	30.2	48	45.3
2003	17	33.3	19	37.3
2007-08	79	46.8	51	30.2
	χ^2 ^= 8.38, p = .02		χ^2 ^= 6.47, p = .04	

When individual newspapers were examined, *The Daily Telegraph *in Sydney had the lowest proportion of positive cycling stories (18.3%), and *The Sydney Morning Herald *had the highest (62.2%), a significant difference (χ^2 ^= 38.59, p < 0.01). In the *Herald Sun *38.3% of stories about cycling were positive compared with 57.1% in *The Age*. When the total number of cycling stories was considered, there were more than twice as many cycling stories in *The Daily Telegraph (n = 104) *as in *The Sydney Morning Herald *(n = 45).

The tabloid newspapers had more negative stories about cycling than the broadsheet newspapers. *The Daily Telegraph *in Sydney had the highest proportion of negative cycling stories (57.7%, n = 60), more than four times that of *The Sydney Morning Herald *(15.6%, n = 7). The *Herald Sun *in Melbourne (n = 36) with 33.6% negative cycling stories, had a higher proportion of negative cycling stories than *The Age *(21.4%, n = 15).

Of the negative cycling stories (n = 122), almost three quarters (73%) were about injury or death to cyclists. The next most common topic was the bad behaviour of the cyclist (20.5% of negative stories). There was variation among the newspapers in how they reported negative cycling stories. There was more frequent reporting of death and injury in the tabloid newspapers (80% of the negative articles in *The Daily Telegraph *and 70.3% in the *Herald Sun*) compared to the broadsheet newspapers (60% of the negative articles in *The Sydney Morning Herald *and 65% in *The Age*).

### News angles

The most common news angle was injury to cyclist(s) (13.2% of articles), followed by death of cyclist(s) (10.7%) (see Table 3). About 10% of the news angles were about moves to support cycling or expressions of support for cycling. This 'support for cycling' news angle became more frequent over time (from 0 in 1998 to 23 in 2008). However, stories about people objecting to cycling or moves to facilitate cycling also rose over time (from 1 in 1998 to 9 in 2008). Cyclists committing misdemeanors on and off the road was the fourth most common news angle (7.4%), however this kind of story fell from 10 in 1998 to 1 in 2008. There were few (4.6%) stories about celebrities riding bicycles, but these became more frequent over time. There were also very few stories about cycle tourism (3.4%), but again these became more frequent. The angle 'cyclists kill pedestrians' appeared to peak around specific events: for example 8 of the 10 stories of this kind appear in the 2007 sample. In a similar fashion, drivers who were charged with killing cyclists gained extra news media attention (with a peak of eight stories of this kind in 2003).

**Table 3:** News angles* by type from 1998 to 2008

	1998	1999	2003	2007	2008	Total	% of total
Injured cyclist(s)	13	9	7	6	8	43	13.2
Killed cyclist(s)	10	1	0	14	10	35	10.7
Supporting cycling	0	0	3	7	23	33	10.1
Misbehaving	10	2	7	4	1	24	7.4
Cycling event	4	2	6	8	1	21	6.4
Driver punished for collision with cyclist	2	4	8	3	0	17	5.2
Celebrity cycling	1	2	2	4	6	15	4.6
Impeding cycling	1	0	1	4	9	15	4.6
Bike facilities improved	3	4	2	5	1	15	4.6
Cycle tourism	1	1	1	3	5	11	3.4
Cyclist kills pedestrian	0	1	1	8	0	10	3.1
Stealing bikes	3	2	1	0	2	8	2.5
Other	13	17	12	16	21	79	24.2
Total	61	45	51	82	87	326	100

* The news angle of a story is the aspect of an issue which triggers the news coverage. It is usually shown in the headline and/or the first paragraph.

### Framing of cycling

As shown in Table 4, the dominant framing of cycling was that cycling is dangerous to cyclists (161 instances, present in 49.4% of cycling articles). One in ten stories also carried the frame that cycling is dangerous to non-cyclists. However, the framing analysis detected that overall there were many more instances of positive framing of cycling than negative (510 vs 308). Analysis of change in the framing over time showed that all positive frames were trending upwards especially 'deserves more support' and 'popular'. While there were fewer instances of negative framing of cycling, all negative framings, except risk to cyclists, were also found to be increasing.

**Table 4:** Positive and negative frames of *cycling *in news stories*

Positive frames	N	%	Negative frames	N	%
Deserves more support	90	27.6	Risk (to cyclists)	161	49.4
Popular	73	22.4	Risk (to non-cyclists)	32	9.8
Quality of life benefits	57	17.5	Negative urban impact	25	7.7
Environmental benefits	51	15.6	Difficult	25	7.7
Promotes health	45	13.8	Unpopular	17	5.2
Fun	43	13.2	Costly	16	4.9
Convenient	38	11.7	Other	32	9.8
Economical	34	10.4			
Other	79	24			

*N = 326, each story may contain more than one frame

The dominant positive frame was that cycling 'deserves more support' (present in 27.6% of articles) (Table 4). This frame is evoked by words and phrases which portray cycling as deserving of community or government support or neglected by society. The frame of cycling being 'popular' was the next most frequently found frame (present in 22.4% of articles), followed by improving 'quality of life' (17.5%) , offers 'environmental benefits' (15.6%), 'promotes health' (13.8%), is 'fun' (13.2%), 'convenient' (11.7%) and 'economical' (10.4%).

The trend for all positive frames was upwards, with the frames of 'deserves more support' and 'popular' increasing more than other positive frames of cycling. 'Quality of life' benefits of cycling showed the next steepest rise, followed by 'environmental benefits' and 'promotes health'. In 1998, positive framing of cycling was dominated by the frames that cycling is 'popular' (11.5% of articles) 'deserves more support' (11.5%) and 'fun' (11.5%). In 1999, the 'deserves more support' (17.8%), 'quality of life' (13.3%) and 'environmental benefits' (11.1%) frames were dominant. In 2003, the dominant positive framings of cycling presented it as 'fun' (17.6%), 'popular' (15.7%) and 'convenient' (13.7%). In 2007, 'popular' (30.5%) rose to the top, followed by 'deserves more support' (26.8%) and 'promotes health' (21.9%). By 2008, the key positives messages were that cycling 'deserves more support' (54%), is 'popular' (32.2%) and confers 'environmental benefits' (32.2%). The year 2008 was distinguished by the presence of the frame of 'deserves more support' in more than half of all articles (54%). The 'promotes health' frame rose from 4.9% in 1998 to a peak of 21.9% in 2007, falling slightly in 2008 to 16.1% when it was overtaken by 'environmental benefits'. 'Environmental benefits' varied between 6.6% and 11.1% until 2008 when almost one third of articles carried this frame (32.2%).

The dominant negative framing of cycling was that it is a 'risk to cyclists' (present in 49.4% of articles) (see Table 4). One in ten articles carried the frame that cycling is a 'risk to non-cyclists'. Framing of cycling as having a 'negative urban impact' or being 'difficult' was present in 7.7% of articles each, and the frames of cycling as 'unpopular' or 'costly' were in 5.2% and 4.9% of articles respectively. About 10% of articles carried other negative framings of cycling.

By far the most dominant negative framing of cycling was 'risk to cyclists'. Although the frequency of this frame fell slightly, this remained by far the most common negative frame. In 1998, cycling was framed negatively as a 'risk to cyclists' (57.4% of articles conveyed this frame) and as 'difficult' (3.3%) and 'unpopular' (3.3%). The frame of 'risk to non-cyclists' was present, but only in 1.6% of articles. By 1999, the negative framing of cycling conveyed the message that it is a 'risk to cyclists' (55.6%) and the proportion of the 'risk to non-cyclists' frame rose to 11.1%. In 2003, the dominant negative framing of cycling was again 'risk to cyclists' (43%), with the 'risk to non-cyclists' framing declining to just 1.9% of articles. In 2007, the dominant negative framing of cycling was 'risk to cyclists' (43.9%), which was followed by 'risk to non-cyclists' (14.6%) and 'negative urban impact' (11%). In 2008, 'risk to cyclists' was still the most widespread negative framing of cyclists (49.4%), while 'negative urban impact' rose to 16% and 'risk to non-cyclists' to 14.9%. The framing of cycling as having a 'negative urban impact' increased the most, from 1.6% in 1998 to 16.1% in 2008.

### Framing of cyclists

The analysis detected remarkably few (n = 58) instances of positive framings of cyclists (as opposed to cycling) (see Table 5). Negative framings of cyclists (n = 107) were detected almost twice as often as positive framings of cyclists. The negative frames of cyclists in this sample paint a picture of cyclists as 'irresponsible lawbreakers', 'pariahs' and 'dangerous to others', a message delivered with greater frequency than the positive presentation of cyclists as 'brave', 'harmless', 'healthy' and 'safety conscious'.

**Table 5:** Positive and negative frames of *cyclists *in news stories*

Positive frames	N	%	Negative frames	N	%
Brave	24	7.4	Irresponsible lawbreakers	38	11.7
Harmless	7	2.1	Pariahs	14	4.3
Healthy	4	1.2	Danger to others	13	4
Safety conscious	3	0.9	Extremists	10	3.1
Other	20	6.1	Inconvenient	9	2.8
			In the minority	6	1.8
			Badly behaved	4	1.3
			Substance abusers	2	0.6
			Other	11	3.4

*N = 326, each story may contain more than one frame

In 1998, one in five (22.9%) cycling articles carried a negative framing of cyclists. These frames were found in 48.9% of articles in 1999, 21.6% in 2003, 33% in 2007 and 38% in 2008. The most dominant frame, 'irresponsible lawbreakers', was present in every year, (between 5 and 11 instances) with a peak of 11 (13.4%) in 2007. Negative portrayals of cyclists developed from 'irresponsible lawbreakers', 'pariahs', 'danger to others', 'inconvenient', 'badly behaved' and 'extremists' in 1998, to 'irresponsible lawbreakers', 'danger to others', 'pariahs', 'inconvenient', 'extremists' and 'in the minority', in 2008.

The proportion of positive framings of cyclists rose from 11.5% of the 61 articles in 1998 to 26.7% in 1999, slumped in 2003 and 2007 (7.8% and 9.8% respectively), and rose to 31% in 2008. In 1998, cyclists were framed as 'brave', 'safety conscious' or 'other', including 'charitable' and 'campaigners for better transport'. In 1999, the 'healthy' frame emerged. In 2008, 'brave' was the leading frame (11 uses), followed by 'harmless' (5) and 'safety conscious' (1). Among the 'other' frames found in 2008 were instances of cyclists as 'image conscious' and as 'outraged by restrictions on taking bikes on trains'.

## Discussion

Over the past decade there has been an increase in the quantity of newspaper coverage of cycling in the two main newspapers in Sydney and Melbourne, with a significant increase in Melbourne in recent years. More recent reporting of cycling has been generally positive, with the Sydney tabloid paper *The Daily Telegraph *reporting the most negative stories. Negative stories were predominately about death or injury to cyclists. Death and injury to cyclists are the two most common news angles, and are the main ways that cycling draws news media attention.

There were large increases in cycling in Melbourne between 2001 and 2006 (a 42% rise from 14,443 to 20,592 people), reported in the Australian Bureau of Statistics journey to work statistics, compared with modest increases in Sydney (8%, from 11,131 to 12,132 people) [1]. This increase in cycling in Melbourne appears to have been followed by positive reporting of cycling in Melbourne. Negative newspaper stories about cycling may deter people from considering cycling as a transport option, but real physical or political improvements to the cycling environment may be necessary before more positive coverage is reported.

Whether the media initiate a widespread change in beliefs and practices in relation to an issue, or react to existing trends (e.g., an upswing in cycling), public health practitioners cannot afford to be passive about news media debates. Most citizens and policymakers learn about health from the media [9-11] and media frames and agendas influence public opinion and attitudes [2,12]. Public health media advocacy, defined as 'the strategic use of news media to advance a public policy initiative, often in the face of opposition' [13] is central to advancing public health [14] and has been critical to policy changes which have reduced death and disability from tobacco, firearms, HIV/AIDS and car accidents [15,16].

The results highlight cycling as an under-appreciated, deserving of support, fun and popular activity which generates social, environmental, health and economic benefits. However, many articles also framed cycling as dangerous to cyclists and non-cyclists and, to a lesser extent, as difficult, bad for the urban environment, unpopular and costly. Given the powerfully negative sentiments sometimes circulating in opinion pages and blogs, it is somewhat surprising to find many more instances of positive frames of cycling than negative. This information is tempered by the findings that almost half of all articles include the frame of cycling as a risk to cyclists and that death, injury and danger were the main ways in which cycling attracted news media attention.

While the proportion of positive framing of 'cyclists' (as opposed to 'cycling') was low, it appears to be rising, with the dominant positive framing of cyclists as 'brave' detected almost four times as frequently in 2008 as in 1998. However, between one-fifth and one-half of the articles carried a negative framing of cyclists in every year studied. While our study shows widespread use of positive framing of cycling, cyclists are portrayed negatively in a substantial subset of coverage (33% on average) and this trend is upwards.

The way that news stories were framed builds a picture of cyclists as irresponsible, law-breaking, dangerous 'others' who behave badly and cause problems for society out of proportion to their numbers. The focus on the label of 'cyclist' tends to connote an image of people who cycle as different from the rest of the population. Perhaps it conjures images of Lycra wearing groups or people wearing fluorescent clothing that is somehow alien to the mainstream, and therefore easier to dismiss or deride. As Koorey [17] says:

'When it comes to cycle planning and policy, all parties involved (politicians, policy-makers, practitioners, advocates) should remember that they are providing for ***"cycling"***, not "cyclists". The former term is an activity that virtually anyone can do under the right circumstances (and hence should be planned for), whereas the latter often gives connotations of a relatively small bunch of "weird" people who only ever cycle.'

Basford and colleagues' [18] study of driver attitudes towards cyclists found that drivers saw cyclists as an "out group", and blamed them accordingly for what was seen as negative behaviour, whilst exonerating members of the "in group", namely themselves and other drivers. Hence, the practice of stereotyping cyclists within the broader culture finds its way into the news media, just as the news media can legitimise and reinforce such stereotypes. Drivers can also be negatively stereotyped by the type of car they drive; four wheel drive vehicles in urban areas have been identified in collective urban mythology, according to Moran, as "over-privileged hoggers of road space", while minicab drivers in England are regarded as "rude, incompetent crooks and potential rapists" [19].

The tendency towards negative framing of cyclists may create barriers to cycling for transport for people who do not identify themselves as in this group. Koorey [17] argues that the majority of people who ride bicycles at least occasionally do not identify themselves as cyclists and for others the term has negative connotations, suggesting that the term 'cyclist' should be avoided in communications promoting cycling to the non-cycling public and motorists. It follows that cycling advocacy should focus more on the benefits of cycling for all, and not focus on the needs of cyclists. Appeals for better cycling infrastructure or policies for the benefit of cyclists may not attract as much public support as cycling changes presented as having benefits for the whole community.

A limitation of the study is that we excluded opinion pieces. They were excluded because they are often written to provoke readers, and do not necessarily reflect popular norms (although do represent some sub-group views). Negative opinion pieces on cyclists in the news have been identified by some cycling advocates as inflaming driver aggression and violence against cyclists. Furthermore, news articles on cycling crashes are more likely to blame and stereotype cyclists as reckless risk-takers rather than develop a more balanced or neutral approach to reporting crashes.

Other limitations include that we did not sample all years (due to resource constraints) or more newspapers. The analysis is restricted to the main Australian cities of Sydney and Melbourne and may not represent newspapers in other capital cities. Excluding sports coverage may have removed a body of positive coverage of cycling. However, cycling for transport and recreation has greater public health importance and was the focus of this study.

## Conclusions

This paper is the first internationally to document how cycling is represented in newspapers and to examine changes in newspaper reporting of cycling over time and by capital city newspapers. Coverage appears to reflect developments in cycling in different locations, but specific newspapers still have particular approaches to how they report stories that range widely on the positive-negative spectrum. Unfavourable news reporting of cyclists and cycling may deter people from considering cycling as a transport option, however physical or political improvements to the cycling environment may be necessary before coverage becomes more positive.

## Competing interests

The authors declare that they have no competing interests.

## Authors' contributions

CR conceived the study, undertook data analysis and lead manuscript preparation.

CB assisted in study design, managed data collection, undertook data analysis, and contributed to manuscript preparation. AE contributed to study design, data analysis, literature reviewing and manuscript preparation. BS undertook re-test reliability analysis and contributed to manuscript preparation. All authors read and approved the final manuscript.

## Pre-publication history

The pre-publication history for this paper can be accessed here:

http://www.biomedcentral.com/1471-2458/10/371/prepub
